# Alzheimer’s Disease Biomarkers Revisited From the Amyloid Cascade Hypothesis Standpoint

**DOI:** 10.3389/fnins.2022.837390

**Published:** 2022-04-27

**Authors:** Deborah O. T. Alawode, Nick C. Fox, Henrik Zetterberg, Amanda J. Heslegrave

**Affiliations:** ^1^Department of Neurodegenerative Disease, UCL Queen Square Institute of Neurology, London, United Kingdom; ^2^UK Dementia Research Institute at UCL, London, United Kingdom; ^3^Department of Neurodegenerative Disease, Dementia Research Centre, UCL Queen Square Institute of Neurology, London, United Kingdom; ^4^Department of Psychiatry and Neurochemistry, Institute of Neuroscience and Physiology, The Sahlgrenska Academy, University of Gothenburg, Gothenburg, Sweden; ^5^Clinical Neurochemistry Laboratory, Sahlgrenska University Hospital, Mölndal, Sweden

**Keywords:** amyloid-beta, blood biomarkers, neurodegeneration, neurofilament light (NfL), glial fibrillary acidic protein (GFAP), phosphorylated tau (p-tau), triggering receptor expressed on myeloid cells 2 (TREM2)

## Abstract

Alzheimer’s disease (AD) is the most common neurodegenerative disease worldwide. Amyloid beta (Aβ) is one of the proteins which aggregate in AD, and its key role in the disease pathogenesis is highlighted in the amyloid cascade hypothesis, which states that the deposition of Aβ in the brain parenchyma is a crucial initiating step in the future development of AD. The sensitivity of instruments used to measure proteins in blood and cerebrospinal fluid has significantly improved, such that Aβ can now successfully be measured in plasma. However, due to the peripheral production of Aβ, there is significant overlap between diagnostic groups. The presence of pathological Aβ within the AD brain has several effects on the cells and surrounding tissue. Therefore, there is a possibility that using markers of tissue responses to Aβ may reveal more information about Aβ pathology and pathogenesis than looking at plasma Aβ alone. In this manuscript, using the amyloid cascade hypothesis as a starting point, we will delve into how the effect of Aβ on the surrounding tissue can be monitored using biomarkers. In particular, we will consider whether glial fibrillary acidic protein, triggering receptor expressed on myeloid cells 2, phosphorylated tau, and neurofilament light chain could be used to phenotype and quantify the tissue response against Aβ pathology in AD.

## Introduction

Alzheimer’s disease (AD) is a neurodegenerative disease characterised by three key neuropathological hallmarks: (1) the presence of amyloid beta (Aβ) plaques in the brain parenchyma, and commonly also within cerebral blood vessels (cerebral amyloid angiopathy); (2) intraneuronal neurofibrillary tangles (NFTs), composed of hyperphosphorylated tau; and (3) neurodegeneration (Stelzmann et al., 1995; DeKosky, 2001; Winner et al., 2011). The accumulation of misfolded proteins, in the form of Aβ plaques and NFTs, has led to AD being termed a proteinopathy (Villemagne et al., 2018). However, of particular interest is the role Aβ plays in AD pathogenesis.

The amyloid cascade hypothesis, described by Hardy and Higgins (1992), introduced the idea of Aβ misfolding and deposition being the primary precipitant of AD, with the other neuropathological hallmarks, including NFT formation and neuronal loss, occurring as a direct consequence of the misfolded Aβ. Along with Aβ misfolding, Hardy and Selkoe (2002) also describe an imbalance between Aβ production and clearance, resulting in an increased presence of cerebral Aβ in its various forms, including monomers, oligomers, insoluble fibrils and plaques (Mawuenyega et al., 2010). Evidence in favour of this hypothesis can be seen in trisomy 21, more commonly known as Down syndrome (DS), as well as in the Icelandic Aβ precursor protein (APP) mutation. DS is caused by individuals having three copies of chromosome 21, the same chromosome that houses the APP gene. It is these additional copies of APP which contribute to DS being the leading genetic cause of AD (Wiseman et al., 2015). Conversely, the A673T point mutation in APP, frequently termed the Icelandic mutation, is the first APP mutation known to be protective against Aβ deposition and AD (Jonsson et al., 2012). This mutation reduces cleavage of APP by β-secretase along its amyloidogenic pathway, and produces Aβ that is less prone to aggregation (Maloney et al., 2014). However, given the multi-factorial nature of AD, it is important to acknowledge that controversies exist regarding the plausibility of the amyloid cascade hypothesis (Morris et al., 2014). Indeed, there is a possibility that the accumulation of by-products of Aβ production, such as the 99 amino acid C-terminal fragment of APP, or even the neuroprotective nature of shorter Aβ peptide fragments, may play a large role in AD pathogenesis (Moore et al., 2018; Pera et al., 2020). Furthermore, there is growing evidence in favour of alternative hypotheses which place other proteins or processes, such as tau and neuroinflammation, as the central initiating mechanisms of AD pathogenesis (Alvarez A. et al., 1999; Alvarez R. et al., 1999; Ittner and Götz, 2011; Guzman-Martinez et al., 2019).

It is now clear that AD neuropathology begins up to 20 years before symptom-onset (Reiman et al., 2012; Villemagne et al., 2013). Furthermore, cerebrospinal fluid (CSF) and plasma Aβ are the first fluid biomarkers to significantly change in AD patients, and they do so prior to Aβ positron emission tomography (PET) positivity (Palmqvist et al., 2019). Therefore, increasing our understanding of this protein is essential to enable us to identify treatments which target AD at its root. In this review, we will consider Aβ deposition as one of the causes of AD, looking at its various isoforms, methods for its detection in biofluids, and biomarkers of tissue reactions to Aβ that could be used as indirect measures of Aβ pathology, and to improve our understanding of Aβ toxicity.

## Amyloid-β Formation, Aggregation and Isoforms

Aβ is a peptide that is naturally present within the healthy human brain, where it is produced intracellularly and at the cell membrane, and subsequently released into the extracellular space (Finder and Glockshuber, 2007). However, pathogenic Aβ is produced when APP, a transmembrane protein, is sequentially cleaved along its amyloidogenic pathway by β- and γ-secretases (Cacace et al., 2016). The site of γ-secretase cleavage determines the length of the resultant Aβ peptide (Perrone et al., 2020), ranging from 37 to 49 amino acids in length (Chen et al., 2017). The most abundant isoforms of Aβ in CSF are Aβ_1–38_, Aβ_1–40_, and Aβ_1–42_ (Struyfs et al., 2015), with the 40 and 42 amino acid length isoforms being the two most widely researched. This is predominantly due to the important role that the Aβ_1–42_/Aβ_1–40_ ratio (Aβ_1–42/1–40_) plays in supporting a diagnosis of AD, as highlighted by the amyloid, tau, neurodegeneration, or AT(N), diagnostic criteria for AD (Jack et al., 2016, 2018). Whilst CSF concentrations of Aβ_1–40_ remain unchanged in AD, Aβ_1–42_ concentrations decrease, which is thought to reflect aggregation and deposition within the brain (Lewczuk et al., 2004). Therefore, looking at the two in combination, as a ratio, provides a more accurate marker of plaque pathology in comparison to the overall Aβ production in that individual, and combats issues of inter-individual baseline concentration differences posed by looking at CSF Aβ_1–42_ concentrations in isolation (Alawode et al., 2021).

However, longer-length peptides, such as Aβ_1–43_, have been observed more frequently than Aβ_1–40_ within Aβ plaque cores in familial AD (FAD), sporadic AD and Down syndrome brains (Hirayama et al., 2003; Welander et al., 2009; Keller et al., 2010). Indeed, post-mortem analysis of AD brains has revealed a positive correlation between Aβ peptide length and plaque load (Aβ_1–43_ > Aβ_1–42_ > Aβ_1–40_) (Jäkel et al., 2019), and studies in mouse models of FAD have revealed that Aβ_1–43_ is more neurotoxic, and has a greater propensity to aggregate, than Aβ_1–42_ (Saito et al., 2011). Furthermore, CSF concentrations of Aβ_1–43_ are significantly reduced in FAD mutation carriers (Perrone et al., 2020), mimicking the reduction in Aβ_1–42_ seen in AD, and highlighting a potential role of Aβ_1–43_ in the disease.

As alluded to above, variability in the C-terminus of Aβ is a well-known phenomenon. However, there is similar heterogeneity at the N-terminus of the peptide (Colin et al., 1985; Miller et al., 1993). In fact, investigations have revealed that only a small proportion of the Aβ ending at amino acids 40 and 42 within cerebral blood vessels and parenchymal plaques, respectively, is made up by Aβ_1–40_ and Aβ_1–42_ (Harigaya et al., 2000), highlighting the overriding presence of truncated or modified Aβ in AD brains. One such truncated species, pyroglutamate-modified Aβ (Aβ_*pE*_), is one of the dominant forms of Aβ in the hippocampi and cortices of AD patients (Portelius et al., 2010), and compared to full-length Aβ, irrespective of the C-terminus, Aβ_*pE*_ that has been truncated and modified at the third amino acid of Aβ (Aβ_*pE*3_) has shown an up to 250-fold increased rate of aggregation (Schilling et al., 2006). Additionally, Aβ_*pE*_ is the only identified form of Aβ that is solely found within plaques, and is not produced by neurones (DeMattos et al., 2012), making it a plaque-specific form of Aβ and a promising immunotherapy target.

Monomeric Aβ exists in both an α-helical and β-pleated sheet conformation, and is amphiphatic in nature, exhibiting hydrophilicity at the N-terminal amino acids, and hydrophobicity at the C-terminus (Finder and Glockshuber, 2007). These monomeric isoforms can subsequently aggregate to form: (1) soluble oligomers, which are heterogenous in size and can spread throughout the brain; (2) protofibrils, which are larger soluble oligomers; or (3) insoluble fibrils, which can further aggregate to form Aβ plaques (Chen et al., 2017; Hampel et al., 2021). All of these aggregated forms of Aβ are known to be neurotoxic (Kuchibhotla et al., 2008; Koffie et al., 2009; Meyer-Luehmann et al., 2009; Hampel et al., 2021). Fibril formation is now widely considered to occur by nucleation-dependent polymerisation (Chatani and Yamamoto, 2018; Pyun et al., 2020). This process involves the initial formation of nuclei, followed by an elongation phase, resulting in fibril formation (Finder and Glockshuber, 2007; Chatani and Yamamoto, 2018). Furthermore, this process is concentration dependent. However, Aβ_1–42_ is much more prone to aggregation, requiring a five-fold lower minimum concentration to aggregate into fibrils than Aβ_1–40_, highlighting why Aβ_1–42_ is much more abundant in plaques than Aβ_1–40_ (Finder and Glockshuber, 2007).

## Tissue Reaction to Amyloid-β Pathology

The presence and accumulation of pathogenic Aβ plaques in the brain parenchyma of AD patients has several neurotoxic effects on the surrounding tissue. Firstly, Aβ triggers an inflammatory response mediated by glial cells within the central nervous system (CNS) (Cai et al., 2014). There is increasing evidence showing that neuroinflammation, and the activation of a variety of CNS-specific glial cells, is emerging as a central player in AD pathogenesis and neuropathology (Heneka et al., 2015; Bronzuoli et al., 2016). As the primary immunosurveillance cells of the CNS, microglia are the first cells to be activated in response to foreign material within the brain. In contrast to microglia, astrocytes function predominantly as a neurosupportive cell type, contributing largely to synaptogenesis, and maintaining synapse and blood-brain barrier (BBB) integrity (Perea et al., 2009; Bronzuoli et al., 2016). When they become activated, microglia secrete pro-inflammatory cytokines, and act as the macrophages of the brain to clear the abnormal debris (Cameron and Landreth, 2009; Ransohoff and Perry, 2009). Furthermore, studies in mouse models of AD have revealed the presence of dystrophic neurites in close proximity to Aβ plaques, which both microglia and astrocytes respond to in an attempt to repair the damage (Sanchez-Varo et al., 2011). Indeed, in the presence of AD neuropathology, both microglia and astrocytes adopt a reactive phenotype, termed reactive gliosis, which has both neuroprotective and neurotoxic effects within AD brains (Bronzuoli et al., 2016). However, the uncontrolled and prolonged activation of both of these glial cells due to increasing Aβ burden eventually leads to their dysfunction, promotes chronic neuroinflammation, and contributes to the loss of synapses around plaques (Canevari et al., 2004; Subramanian et al., 2020).

Secondly, the presence of Aβ induces oxidative stress and disrupts calcium homeostasis, leading to neuronal toxicity (Canevari et al., 2004). This toxicity results in necrotic neuronal death, and the resultant decrease in brain volume associated with AD, particularly in the hippocampi (Behl et al., 1994). Along with the loss of synapses, this neurodegeneration significantly contributes to the cognitive decline seen in AD patients (Deture and Dickson, 2019).

Thirdly, Aβ induces the intraneuronal phosphorylation of tau (a protein that is microtubule-associated in its non-phosphorylated state), its subsequent aggregation to form NFTs, and its extracellular secretion (Busciglio et al., 1995; Mattsson-Carlgren et al., 2020). Tau phosphorylation, which may eventually cause NFT pathology, disrupts the neuronal cytoskeleton, and similar to Aβ, contributes to synapse loss and cognitive decline (Gómez-Isla et al., 1997; Metaxas and Kempf, 2016).

In essence, Aβ pathology, and the resultant tissue reactions to this peptide, are known processes that are now possible to monitor and measure in biofluids as well as with PET neuroimaging. This has been made possible due to the recent developments in supersensitive immunoassay and mass spectrometry (MS) detection methods.

## Methods for Fluid Biomarker Measurement

Methods for fluid biomarker measurement have been around for many years. With respect to Aβ and its tissue reactions, they have largely involved the use of enzyme-linked immunosorbent assay (ELISA) (Sjögren et al., 2001; Welge et al., 2009; Ishiki et al., 2016; Piccio et al., 2016). However, as we move into measuring Aβ and markers of its tissue reactions in blood, there is a need to use more sensitive detection methods. Hence, for the purposes of this review, we will focus on Single molecule array (Simoa) technology and MS.

### Single Molecule Array

Simoa is a form of digital ELISA that measures the fluorescence from single enzyme-labelled protein molecules conjugated onto magnetic beads, and trapped within femtolitre-sized wells (Rissin et al., 2010). Whilst conventional ELISA is currently the gold standard technique for detecting and quantifying proteins, digital ELISA using Simoa is emerging as an ultrasensitive method for protein detection, increasing sensitivity of protein detection from picomolar (10^–12^ M) to subfemtomolar (10^–15^ M) concentrations (Rissin et al., 2010; Cohen and Walt, 2019; Kan et al., 2020). This is particularly important as the serum concentration of proteins involved in AD are thought to range from 10^–16^ to 10^–12^ M (Galasko, 2005; Jong et al., 2007). Simoa measurements of Aβ, glial fibrillary acidic protein (GFAP), phosphorylated tau (p-tau) and neurofilament light chain (NfL), but not the soluble fragment of triggering receptor expressed on myeloid cells 2 (sTREM2), in both CSF and plasma are now well-established (Janelidze et al., 2016; Hendricks et al., 2019; Vergallo et al., 2019; Benedet et al., 2021; Blennow, 2021; Park et al., 2021). However, as we move into the era of disease-modifying therapies for AD targeting Aβ pathology, there is a need for more sensitive and robust techniques to measure changes in these proteins in trial participants. Furthermore, a more sensitive detection method provides the possibility of diluting samples prior to analysis, thereby reducing matrix effects without compromising detectability of proteins of interest (Song et al., 2015). One possible solution to this ongoing issue of sensitivity is the recent development of upgraded Simoa technology, which can measure proteins down to sub-attomolar (10^–18^ M) concentrations (Kan et al., 2020). This increased sensitivity has been achieved primarily by: (1) increasing the molecule:bead ratio through the use of fewer beads (5,000 compared to 500,000 in conventional Simoa); and (2) using magnetic-meniscus sweeping to increase the proportion of beads loaded into the microwells (Kan et al., 2020). Although this upgrade is not yet commercially available, preliminary investigations have revealed an increase in sensitivity of up to 100-fold provided by this technique compared to conventional Simoa (Kan et al., 2020).

### Mass Spectrometry

MS is an analytical technique for measuring the mass-to-charge ratio of analyte ions, thus, by nature, it can achieve a greater specificity compared to immunoassays. Standard MS workflow involves an initial separation step of the analyte prior to analysis and detection within the mass spectrometer. Various systems can be coupled to the mass spectrometer in order to achieve this separation, including liquid chromatography and gas chromatography (Smith, 2013). One key difference compared to immunoassays is that the mass spectrometer is antibody-independent. This can be important where there are no suitable antibodies for detection purposes, although antibodies can be utilised to enrich samples by immunoprecipitation prior to the mass spectrometry step. Denaturing conditions, used in sample preparation, as well as the samples being handled in aqueous organic solvents, mean results obtained can be less influenced by matrix effects than in an immunoassay (Pannee et al., 2014; Crutchfield et al., 2016; Oeckl and Otto, 2019).

Arguably of most interest with regards to Aβ is a direct comparison of MS with Simoa-based quantification of Aβ_1–40_ and Aβ_1–42_ in a preclinical AD cohort, conducted by Keshavan et al. (2021), which observed that at this stage of disease, MS measurements showed a higher correlation with brain Aβ pathology than Simoa measurements. Further comparisons between MS and other immunoassay techniques have shown similar results. A head-to-head comparison of eight plasma Aβ_1–42/1–40_ assays, including four MS and four immunoassays, revealed that MS methods for plasma Aβ_1–42/1–40_ measurement provide greater discriminative accuracy between Aβ-positive and Aβ-negative individuals, as measured by Aβ PET (Janelidze et al., 2021). Furthermore, MS correlates better with CSF Aβ_1–42/1–40_ measurements than immunoassay methods in two independent disease cohorts (Janelidze et al., 2021). In particular, immunoprecipitation-MS developed by Randall Bateman at Washington University was observed as the most superior in all aspects assessed. The promising results observed with MS in comparison to immunoassays opens questions as to whether MS may be the future of Aβ measurements. Furthermore, MS methods for detection of p-tau, and sTREM2 have also shown promise (Heslegrave et al., 2016; Korecka and Shaw, 2021), with little to no published work on MS detection of GFAP or NfL. However, it is important to consider the space, cost and sample preparation time required for a mass spectrometer, and whether the differences between MS and immunoassays are significant enough to warrant a complete change to purely MS-based analyses of these proteins.

## Apparent Fluid Biomarkers for Amyloid-β Pathology

Historically, AD diagnoses were made based on symptoms alone. However, studies have revealed that the neuropathological hallmarks of AD begin up to 20 years prior to symptom onset (Reiman et al., 2012; Villemagne et al., 2013). As such, there has been a move towards using a biological definition of disease to support a clinical diagnosis of AD (Jack et al., 2018). The AT(N) criteria for AD diagnosis draws together the three key pathophysiological characteristics of AD using both fluid and neuroimaging biomarkers, and is described by Jack et al. (2016). However, for the purposes of this review, we will focus solely on the fluid biomarkers of Aβ pathology. In particular, we will consider direct fluid biomarkers of Aβ pathology – Aβ_1–42/1–40_ and Aβ_1–43_ – and markers of tissue reactions to Aβ – GFAP, triggering receptor expressed on myeloid cells 2 (TREM2), p-tau, and NfL.

### Direct Markers of Amyloid-β Pathology

#### Amyloid-β 1–40, 1–42, 1–42/1–40 and 1–43

As highlighted by Jack et al. (2016), the fluid biomarkers recognised by the AT(N) criteria for “A” or Aβ pathology are CSF Aβ_1–42_ or Aβ_1–42/1–40_. To place an individual on the AD continuum, they must have evidence of positive Aβ pathology, whether that be through a reduction in the CSF biomarkers or a positive Aβ PET scan. The absence of positive Aβ biomarkers is suggestive of a non-AD pathological change (Jack et al., 2018). The recent update of the AlzBiomarker database comprises a meta-analysis of studies comparing biomarker levels in AD vs controls, as well as cross-disease comparisons (Accessed 7th December 2021)^1^ (Olsson et al., 2016). The database reveals that in AD vs controls, CSF Aβ_1–40_ decreases by 9%, whereas CSF Aβ_1–42_ decreases by 45%, highlighting a much larger decrease in CSF Aβ_1–42_ in AD. Both of these percentage decreases are statistically significant.

Studies on CSF concentrations of Aβ_1–43_ have revealed this peptide, as well as CSF Aβ_1–43/1–40_, to be significantly reduced in both AD compared to controls and early-onset AD compared to late-onset AD (Lauridsen et al., 2017; Perrone et al., 2020). However, in contrast to Aβ_1–40_ and Aβ_1–42_, the quantity of studies investigating CSF Aβ_1–43_ remains relatively scarce. One possible reason for this is that some studies have observed little difference between the diagnostic accuracy of Aβ_1–42_ and Aβ_1–43_ (Bruggink et al., 2013; Lauridsen et al., 2017). Therefore, it could be argued that given the already established robustness of CSF Aβ_1–42_ and Aβ_1–42/1–40_ in AD diagnosis, there is no place for Aβ_1–43_. However, one study found CSF Aβ_1–43_, but not CSF Aβ_1–42_, could identify patients with amnestic MCI who later progressed to AD, as well as observing a significant decrease in CSF Aβ_1–43_ concentrations over the 2-year follow-up period, compared to no significant differences in CSF Aβ_1–42_ concentrations (Lauridsen et al., 2016). Furthermore, a separate study observed CSF Aβ_1–43_ concentrations to be significantly reduced to a greater extent in early onset AD compared to late onset AD – a finding not true to CSF Aβ_1–42_ (Lauridsen et al., 2017). Together, these studies highlight a potential role of Aβ_1–43_, albeit less well investigated, in distinguishing between AD diagnostic groups (Alawode et al., 2021).

Aβ PET has been used to assess the presence or absence of Aβ pathology for several years, and was used to validate the sensitivity and efficacy of the CSF Aβ biomarkers (Blennow et al., 2015). Whilst the ability to detect the presence of Aβ pathology using CSF has been invaluable, there is a need to develop blood-based biomarkers for diagnosis. This is because blood measurements incur much lower costs and are more easily accessible in low-resource and non-specialist settings worldwide (Molinuevo et al., 2018; Shi et al., 2018; Albani et al., 2019). Additionally, blood can be obtained less invasively than CSF, so could function as an initial diagnostic screening tool in the primary care setting, followed by more in-depth analysis in specialist centres (Janelidze et al., 2016; Molinuevo et al., 2018). However, drawbacks evidently exist in measuring biomarkers of brain diseases using peripheral body fluids, namely: (1) the BBB results in a 10–100 fold lower concentration of the analytes compared to in CSF (Blennow and Zetterberg, 2015); (2) some AD biomarkers are expressed by extra-cerebral tissues; (3) analytes of interest may undergo degradation by blood proteases prior to their measurement (Zetterberg and Burnham, 2019). Indeed, whilst Aβ is most commonly discussed with regards to AD, Aβ production appears to occur in all cells and tissues of the body (Li et al., 1999), and deposits have been observed in extra-cerebral tissues, including systemic blood vessels, platelets, skin, subcutaneous tissue, intestines and the eye (Mori et al., 1989; Li et al., 1999; Hart et al., 2016). Plasma and serum Aβ_1–40_, Aβ_1–42_, or Aβ_1–42/1–40_ measurements have been investigated as potential blood biomarkers for AD. However, despite plasma Aβ concentrations fluctuating with time, continuous contributions from extra-cerebral tissues mean that plasma Aβ concentrations do not change as dynamically in AD as CSF Aβ (Roher et al., 2009; Palmqvist et al., 2019). This is highlighted in the AlzBiomarker database, which reveals that in contrast to the decreases in CSF Aβ concentrations, which is expected as this reflects Aβ aggregation into plaques, plasma Aβ_1–40_ increases by 4%, and plasma Aβ_1–42_ increases by 5%. The marginal increases in these biomarkers in plasma, as well as the lower overall degree of change observed, is most likely a consequence of the peripheral production of Aβ, which is unaffected by pathology. This highlights the need to develop and measure biomarkers of Aβ pathology which can better distinguish between AD and non-AD individuals, particularly in blood, but which also better reflect the pathology occurring within the brains of AD patients. This is where looking at the markers of tissue reaction to Aβ may prove useful.

### Markers of Tissue Reaction to Amyloid-β

The deposition of Aβ within the brain parenchyma results in numerous effects on the surrounding tissue, including glial cell activation and neuroinflammation, tau phosphorylation, and neurodegeneration, as already discussed. Increasing evidence is showing that it may be possible to use markers of these processes as indirect markers of cerebral Aβ pathology.

#### Glial Fibrillary Acidic Protein

GFAP is a well-known marker of astrocytosis in the CNS. Early *in vivo* studies observed Aβ-containing astrocytes in the brains of AD patients (Funato et al., 1998; Thal et al., 2000). A subsequent *in vitro* investigation revealed that astrocytes can phagocytose Aβ (Wyss-Coray et al., 2003), which is the most likely cause of the intracellular Aβ observed in the two aforementioned *in vivo* studies. Whilst the exact role of astrocytosis in AD remains unclear, it is apparent that reactive astrocytes follow the same spatial distribution as plaques in post-mortem analyses of AD brains (Beach and McGeer, 1988; Perez-Nievas and Serrano-Pozo, 2018). Furthermore, investigations have revealed that reactive astrocytes are involved in Aβ production and toxicity (Garwood et al., 2011; Söllvander et al., 2016). It was previously thought that the number of astrocytes surrounding plaques increases as the disease progresses (Pike et al., 1995; Vehmas et al., 2003). However, more recent studies using a combination of PET tracers have revealed that astrocytosis (depicted by the ^11^C-deuterenium-L-deprenyl tracer), is an early phenomenon in AD (depicted by the ^11^C-Pittsburgh compound-B tracer for Aβ plaques), and this astrocytosis decreases as plaque load increases (Carter et al., 2012; Scholl et al., 2015; Rodriguez-Vieitez et al., 2016).

Studies have shown that CSF GFAP concentrations in AD are significantly increased compared to healthy controls (Colangelo et al., 2014; Ishiki et al., 2016), and are significantly increased in the cognitively unimpaired Aβ-positive, tau-positive preclinical stage of AD (Milà-Alomà et al., 2020). However, cross-disease comparisons between AD, frontotemporal lobe dementia (FTLD) and dementia with Lewy bodies (DLB) reveal that CSF GFAP concentrations are significantly increased in all three diseases compared to controls, with FTLD concentrations being significantly greater compared to AD and DLB (Ishiki et al., 2016). This highlights that elevated CSF GFAP is not specific to AD, and hence has little diagnostic value in distinguishing AD from other neurodegenerative diseases.

Interest in GFAP as a plasma biomarker for AD came about due to the possibility of more sensitive assays making it possible to measure within blood. Similar to CSF GFAP, elevated plasma GFAP concentrations have been observed in a variety of neurodegenerative and non-neurodegenerative neurological conditions, including AD (Mayer et al., 2013; Elahi et al., 2020; Heller et al., 2020; van Ballegoij et al., 2020). However, further investigations have revealed that plasma GFAP concentrations correlate strongly with cerebral Aβ pathology, as measured by PET (Verberk et al., 2020), as well as with decreasing white matter volume and worsening cognitive function (Oeckl et al., 2019; Rajan et al., 2020; Verberk et al., 2020; Asken et al., 2021), and hence it is relatively Aβ-specific. In fact, simultaneous comparisons in two independent cohorts between plasma GFAP and NfL, a sensitive biomarker of neuronal injury independent of Aβ pathology, revealed that plasma GFAP may be more sensitive to cortical and cognitive changes than plasma NfL (Asken et al., 2021). Plasma GFAP is higher in Aβ-positive cognitively unimpaired individuals at risk of developing AD (Chatterjee et al., 2021), and longitudinal investigations have observed that plasma GFAP can predict subsequent conversion of mild cognitive impairment (MCI) patients to AD with an area under the receiver operating characteristic curve of 0.84 (95% CI 0.77–0.91) (Cicognola et al., 2021). Furthermore, individuals with a positive CSF Aβ_1–42/1–40_, but with Aβ PET levels below the cut off for being deemed Aβ PET-positive (i.e., individuals in the earliest preclinical stage of AD), were observed to have significantly higher plasma GFAP concentrations than Aβ-negative individuals, despite there being no significant difference in CSF GFAP concentrations between the two groups (Benedet et al., 2021). One possible reason for this is that GFAP may be released more directly into the bloodstream by astrocytic end feet, thus making plasma changes in GFAP concentrations more pronounced than changes in CSF GFAP concentrations (Giannoni et al., 2018). This is further supported by a plethora of evidence highlighting that the integrity of the BBB is abnormal in AD, resulting in microvascular leakage of proteins into the blood (Banks, 2012). Another reason for significant increases in plasma GFAP, but not CSF GFAP, in AD patients may be due to GFAP being extremely stable in blood, whereas CSF GFAP is much more sensitive to freeze-thaw cycles overtime (Abdelhak et al., 2019; Ashton et al., 2021). However, further work must be undertaken to better understand the reason for this discrepancy between plasma and CSF GFAP. Nonetheless, together these studies highlight that astrocytosis begins in the prodromal stage of AD, and elevated plasma GFAP is associated with neuronal injury, worsening cognition, and markers of cerebral Aβ pathology. Whilst plasma Aβ_1–42/1–40_ may also function well as a plasma biomarker of Aβ pathology, plasma GFAP appears to give a much broader picture of the state of the individual, hence it may better function as a surrogate plasma biomarker for Aβ pathology in AD. This is further supported by a 50% increase in CSF GFAP compared to a 93% increase in plasma (see text footnote 1; Accessed 7th December 2021) in AD vs controls, with only the plasma GFAP changes being statistically significant.

#### Triggering Receptor Expressed on Myeloid Cells 2

As discussed, neuroinflammation is emerging as a central player in AD pathogenesis and neuropathology (Bronzuoli et al., 2016). In response to Aβ, microglia upregulate the expression of TREM2, an innate immune transmembrane receptor expressed by myeloid cells. Within the CNS, TREM2 is unique to microglia, however, peripherally it is also expressed by immature dendritic cells and osteoclasts (Neumann and Takahashi, 2006; Jiang et al., 2014; Ghosh et al., 2021). TREM2 is required to initiate and promote microglial activation and Aβ phagocytosis (Heneka et al., 2015; Wang et al., 2015), and it plays an essential role in maintaining CNS homeostasis by mediating the phagocytic function of microglia, suppressing pro-inflammatory cytokine release and enhancing transcription of anti-inflammatory cytokines (Neumann and Takahashi, 2006; Paradowska-Gorycka and Jurkowska, 2013). This is further highlighted by a homozygous loss of function mutation in the TREM2-encoding gene resulting in a neurological disease characterised by: (1) chronic neurodegeneration, most likely caused by the decreased clearance of debris by microglia, and a resultant increase in proinflammatory cytokines and neuroinflammation; and (2) bone cysts due to abnormal osteoclast maturation and function (Paloneva et al., 2001, 2002; Neumann and Takahashi, 2006). Furthermore, early AD studies have revealed that activated microglia concentrate around Aβ deposits in both AD patients and mouse models of AD, highlighting the role receptors must play in mediating this interaction (Dickson et al., 1993; Paresce et al., 1996; Frautschy et al., 1998). In particular, the discovery that a heterozygous missense mutation in the TREM2-encoding gene is a risk factor for development of AD, to a similar extent as that observed for apolipoprotein ε4, highlights the role TREM2, and hence microglia, play in AD neuropathology (Guerreiro et al., 2013; Jonsson et al., 2013; Slattery et al., 2014; Rosenthal et al., 2015).

When TREM2 is proteolytically cleaved, sTREM2 is produced and can be measured in CSF to assess TREM2 activity, and hence act as a surrogate marker for microglial activation (Ulrich et al., 2017). Attempts to measure CSF sTREM2 concentrations in AD have shown significantly higher concentrations in pre-symptomatic AD, MCI, and early/mild AD (Henjum et al., 2016; Heslegrave et al., 2016; Piccio et al., 2016; Suarez-Calvet et al., 2016). As the disease progresses, CSF sTREM2 concentrations appear to decrease, such that there is either no significant difference, or a significant reduction, in AD compared to controls (Kleinberger et al., 2014; Henjum et al., 2016; Suarez-Calvet et al., 2016). This highlights a longitudinal change in CSF sTREM2, with concentrations peaking in MCI and progressively decreasing as disease severity increases (Suarez-Calvet et al., 2016). Interestingly, CSF sTREM2 concentrations have been found to correlate with CSF total tau (t-tau) and p-tau, but not with CSF Aβ_1–42_ (Heslegrave et al., 2016; Piccio et al., 2016). This suggests that although TREM2 correlates with neuronal injury and tau pathology markers, it may not reflect Aβ pathology, which is further highlighted by elevated CSF sTREM2 levels in the absence of Aβ pathology (Suarez-Calvet et al., 2016). Evidence has shown that Aβ deposition and neuronal injury precede elevated CSF sTREM2 concentrations in pre-symptomatic patients (Suárez-Calvet et al., 2016). Therefore, it is possible that as the disease progresses, there is a failure of microglial function, particularly in phagocytosing Aβ plaques, and hence reduced TREM2 activity. Alternatively, TREM2 may be a marker of Aβ-induced tau pathology and neurodegeneration in AD (Park et al., 2021), supporting the notion that microglial activation may drive tau pathology (Pascoal et al., 2021).

Unlike CSF sTREM2, where there is a 31% increase in AD compared to controls (see text footnote 1; Accessed 7th December 2021), no significant difference has been observed in plasma sTREM2 concentrations (Kleinberger et al., 2014; Piccio et al., 2016; Bekris et al., 2018). Furthermore, plasma sTREM2 concentrations do not correlate with CSF t-tau, p-tau, or Aβ_1–42_ (Piccio et al., 2016; Bekris et al., 2018). Interestingly, Bekris et al. (2018) observed a significant positive correlation between plasma and CSF sTREM2, whilst Piccio et al. (2016) observed a non-significant positive correlation between plasma and CSF sTREM2 concentrations, and Park et al. (2021) observed a negative correlation between plasma and CSF sTREM2. The cause of the differing results between these studies is not clear but may be due to differences in cohort sizes or methodological factors. A longitudinal study found elevated serum sTREM2 constituted an increased risk of dementia (Ohara et al., 2019). However, the criteria used by Ohara et al. (2019) to diagnose AD within their patient cohort were purely clinical, with no biomarker evidence of AD assessed, therefore it is possible that some patients were misdiagnosed with AD. Whilst sTREM2 is measurable in both plasma and serum, direct comparisons of absolute levels in these matrices, to our knowledge, have not been performed. Overall, the general consensus appears to be that plasma sTREM2 may not be a useful biomarker for use in AD, particularly with regards to tissue reactions to Aβ. This is because plasma sTREM2 can reflect peripheral inflammation produced by other cells of myeloid origin, in contrast to CSF sTREM2, which is microglia-specific (Rodriguez-Vieitez and Ashton, 2021).

#### Phosphorylated Tau

Similar to Aβ, tau is a natural component of mature neurones, with some healthy individuals also having a small percentage of p-tau, as phosphorylation appears to be important in enabling the normal function of tau within neurones (Barthélemy et al., 2020). However, in AD, tau is 3–4 fold more phosphorylated, and it is this hyperphosphorylation that promotes the intraneuronal aggregation of tau into NFTs (Vanmechelen et al., 2000; Parnetti et al., 2001; Buerger et al., 2002). However, as well as aggregating intraneuronally, p-tau is secreted from neurones, and can subsequently be measured in CSF and blood. In fact, it is possible that CSF changes in p-tau occur prior to NFT formation (Barthélemy et al., 2020). There are up to 85 sites at which tau can be phosphorylated (Martin et al., 2011), with the three most widely investigated sites in relation to AD being tau phosphorylated at threonine 181 (p-tau_181_), threonine 217 (p-tau_217_), and threonine 231 (p-tau_231_). In contrast to t-tau, which is included in the AT(N) criteria for AD diagnosis as a general marker of neurodegeneration and neuronal injury, there is no change in CSF p-tau concentrations in other tauopathies and neurological conditions (Hesse et al., 2001; Parnetti et al., 2001; Riemenschneider et al., 2003). Rather, CSF p-tau is significantly increased in AD compared to controls and other neurodegenerative diseases, regardless of which epitope is measured (Kohnken et al., 2000; Vanmechelen et al., 2000; Buerger et al., 2002; Verbeek et al., 2004; Welge et al., 2009). However, when combined with increased CSF p-tau, increased CSF t-tau does indeed reflect AD pathology, as opposed to being a general indication of neuronal injury (Alawode et al., 2021). Comparing and contrasting p-tau_181_, p-tau_217_, and p-tau_231_ is beyond the scope of this review, so we will look at p-tau in general. However, the analytical and clinical performance of assays detecting these three tau epitopes was recently assessed by Bayoumy et al. (2021).

In light of CSF p-tau being significantly increased in AD compared to controls, several studies have shown a clear correlation between CSF p-tau and Aβ pathology measures (Bateman et al., 2012; Barthélemy et al., 2020; Suárez-Calvet et al., 2020), with changes in CSF p-tau also being observed several years prior to symptom onset, and when only subtle changes in Aβ pathology measures are detected. Furthermore, CSF p-tau has been shown to correlate more strongly with cognitive impairment than Aβ biomarkers (Gómez-Isla et al., 1997; Nelson et al., 2012; Jack et al., 2018). Given that increases in CSF p-tau are unique to AD, and are not observed in other tauopathies, it is hypothesised that p-tau may be a measure of Aβ-induced tau phosphorylation (Maia et al., 2013; Kanmert et al., 2015; Sato et al., 2018). Furthermore, CSF p-tau_181_, p-tau_217_, and p-tau_231_ exhibit remarkably high increases of 87, 999, and 489%, respectively, in AD compared to controls (see text footnote 1; Accessed 7th December 2021). Given the overwhelming evidence showing CSF p-tau to be a robust biomarker for AD, the question lies with whether plasma p-tau correlates as strongly with Aβ pathology, and whether there is scope for plasma p-tau to function as a biomarker of Aβ pathology better than Aβ biomarkers.

Attempts to quantify plasma p-tau began in 2016 and have proven largely successful. Similar to CSF p-tau, plasma p-tau has been found to increase in AD compared to MCI, non-AD dementias and cognitively unimpaired controls (Shekhar et al., 2016; Tatebe et al., 2017; Janelidze et al., 2020; Karikari et al., 2020b). In particular, Mielke et al. (2018) showed that plasma p-tau is strongly associated with Aβ PET imaging, and is highly sensitive and specific to increased cerebral Aβ burden, whilst Karikari et al. (2020a) showed that plasma p-tau increases markedly in Aβ PET negative individuals who also have decreased CSF Aβ concentrations. Furthermore, in their cross-sectional study looking at biomarker trajectories with increasing Aβ burden, Palmqvist et al. (2019) showed that in AD plasma p-tau changes significantly before CSF and plasma Aβ_1–42/1–40_, and CSF p-tau, all of which exhibit changes before Aβ PET positivity is detected. In addition, they showed that plasma p-tau continues to increase as Aβ burden increases (Palmqvist et al., 2019). This highlights that plasma p-tau may be one of the earliest biomarkers to change in AD, and it continues to reflect Aβ pathology whilst also giving additional information on the progression of tau pathology up to 10 years before tau PET positivity is detected (Bateman et al., 2012). This is further highlighted by increases in plasma p-tau_181_ and p-tau_217_ of 80 and 288%, respectively, in AD compared to controls (see text footnote 1; Accessed 7th December 2021).

#### Neurofilament Light Chain

Along with microtubules and microfilaments, neurofilaments form the neuronal cytoskeleton (Yuan and Nixon, 2021). However, one particular subunit, NfL, is expressed predominantly in large-calibre myelinated axons (Schlaepfer and Lynch, 1977). Following neuronal damage and degeneration, NfL is released into the extracellular space, and is detectable both in CSF and in blood (Lista et al., 2017). Therefore, by proxy, biofluid changes in NfL are not specific to AD, but reflect general neuronal death and axonal loss. Nonetheless, CSF NfL is significantly increased in AD compared to controls, and predicts progression from MCI to AD (Sjögren et al., 2001; Petzold et al., 2007; Mattsson et al., 2016; Olsson et al., 2016; Zetterberg et al., 2016; Lista et al., 2017). Of particular interest for this review is whether CSF NfL correlates with Aβ pathology. A study by Zetterberg et al. (2016) observed that whilst there were correlations between increased CSF NfL and decreased CSF Aβ_1–42_, there was no significant difference in CSF NfL concentrations between the Aβ-positive and Aβ-negative groups. This has been further corroborated by several studies (Jin et al., 2019; Aschenbrenner et al., 2020; Dhiman et al., 2020). Interestingly, Dhiman et al. (2020) observed that a combination of CSF NfL and a ratio between NfL and Aβ_1–42_ (NfL/Aβ_1–42_) predicted Aβ burden, brain atrophy and altered cognition. Nonetheless, these studies highlight that changes in CSF NfL occur independently of Aβ pathology, therefore CSF NfL is not suitable as a surrogate biomarker for Aβ pathology in AD. Rather, evidence suggests that CSF NfL correlates better with tau biomarkers (Olsson et al., 2016; Zetterberg et al., 2016; Dhiman et al., 2020).

Overwhelming evidence has shown that not only does plasma NfL significantly increase in AD compared to controls, but it also correlates with CSF NfL and tau biomarkers (Mattsson et al., 2017; Lewczuk et al., 2018; Lin et al., 2018). Mattsson et al. (2017) and Lewczuk et al. (2018) both observed a correlation between increased plasma NfL and decreased CSF Aβ_1–42_. However, when this correlation was investigated further, Lewczuk et al. (2018) found it to no longer be significant when the diagnosis of each individual was taken into consideration – a finding supported by Sanchez-Valle et al. (2018). Although increases in plasma NfL are not unique to AD, and can be seen in several other neurodegenerative and non-neurodegenerative conditions (Gisslén et al., 2016; Rohrer et al., 2016; Rojas et al., 2016; Weydt et al., 2016), a recent longitudinal study revealed that plasma NfL is increased up to 22 years prior to expected AD symptom onset (Quiroz et al., 2020), which is consistent with earlier studies of serum NfL in FAD (Preische et al., 2019; Weston et al., 2019). With very little difference between plasma and CSF NfL increases in AD compared to controls (98% in CSF vs 85% in plasma; see text footnote 1; Accessed 7th December 2021) it is clear that plasma NfL may play a more advantageous role in AD than CSF NfL. However, neither plasma nor CSF NfL measures correlate with Aβ neuropathology, hence are not suitable for use as surrogate biomarkers for Aβ.

## Conclusion

In this manuscript, we have considered Aβ as one of the causes of AD, as described in the amyloid cascade hypothesis, and how this can be detected and monitored using biofluid-based biomarkers. Furthermore, we have investigated the evidence supporting and opposing four key biomarkers of tissue reactions to Aβ being used as indirect biomarkers of Aβ pathology in AD diagnosis and clinical trials – GFAP, TREM2, p-tau, and NfL. Up until now, Aβ PET was the gold standard for confirming the presence of Aβ pathology in clinical trials. CSF Aβ measurements have recently been used in conjunction with Aβ PET and have proven to detect pathology at an earlier stage. However, we now have access to sensitive tests for plasma Aβ, which have revolutionised the field. There is now a move towards blood-based biomarker measurements in AD due to blood being cheaper and less invasive to obtain. However, the peripheral production of Aβ makes measuring this protein in blood, and distinguishing between AD, non-AD and controls, particularly challenging, as there is significant overlap in plasma Aβ concentrations between these groups. Therefore, finding an alternative biomarker to measure Aβ pathology indirectly is extremely advantageous for improving diagnostic accuracy, monitoring disease progression, and assessing response to treatment. With regards to sensitivity and specificity, for some biomarkers, including Aβ_1–42/1–40_, diagnostic accuracy for CSF measurement is much greater than that of blood measurements (Palmqvist et al., 2020). Whereas, some biomarkers perform as well in blood as in CSF, such as p-tau_217_ (Palmqvist et al., 2020).

Of the four tissue response biomarkers discussed, plasma GFAP appears to be the most promising indirect biomarker of Aβ pathology. In particular, it gives a broader picture of the pathological state of the individual, also providing additional information on neuronal injury and cognitive decline, as well as highlighting astrocyte response to AD pathology. As such, it would be a useful biomarker for preliminary AD diagnosis, and in clinical trials for pre-screening purposes. Furthermore, in relation to drugs targeting Aβ, plasma GFAP could provide useful information on drug efficacy and therapy response, as if Aβ is decreasing successfully with the use of the drug, the tissue reaction to Aβ should also decrease. Whilst plasma p-tau also reflects Aβ pathology, and it does so earlier than plasma and CSF Aβ measures, plasma p-tau would serve better as an early marker of tau pathology, in particular to confirm the presence of Aβ-induced tau phosphorylation. Additionally, it could function well as a therapy response marker. In contrast, plasma sTREM2 shows no significant difference in AD compared to controls, so has no evident role in AD diagnosis and clinical trials at this stage. Finally, whilst plasma NfL does correlate with markers of tau pathology and neurodegeneration, it shows no correlation with markers of Aβ pathology. Nonetheless, it is an excellent biomarker of neuronal injury, so could function in combination with other AD pathology markers to confirm the presence of neurodegeneration, and as a dynamic biomarker, it can also be used to monitor treatments.

One thing that has not been discussed is the role these biomarkers may play in personalised medicine approaches to AD treatment. Whilst the breadth of licensed drugs for AD treatment is currently relatively small, the biomarkers of tissue reactions to Aβ could give further insight into which disease pathways appear to be most active in individual patients, which in itself will provide valuable information on which disease pathways should be specifically targeted within that patient. This may pave the way for a repurposing of drugs currently used to treat other non-AD pathologies. It is clear that biomarkers of tissue reactions to Aβ have a role to play in AD research and clinical practice, whether that be in diagnosis, clinical trials and/or treatment. The recent advances in measurements of blood biomarkers that reflect brain pathology, including Aβ-specific reactions, will enable large scale screening for trial recruitment, meaning that a move towards blood-based biomarkers for AD will be key for this rapidly changing field.

## Author Contributions

DA wrote the manuscript. All authors contributed to the concept and content of review, manuscript revision, read, and approved the final version.

## Conflict of Interest

NF served as a consultant, at advisory boards, or on a data monitoring committee for Roche, Biogen, and Ionis. HZ served at scientific advisory boards and/or as a consultant for Abbvie, Alector, Annexon, Artery Therapeutics, AZTherapies, CogRx, Denali, Eisai, Nervgen, Pinteon Therapeutics, Red Abbey Labs, Passage Bio, Roche, Samumed, Siemens Healthineers, Triplet Therapeutics, and Wave, has given lectures in symposia sponsored by Cellectricon, Fujirebio, Alzecure, Biogen, and Roche, and was a co-founder of Brain Biomarker Solutions in Gothenburg AB (BBS), which is a part of the GU Ventures Incubator Program. The remaining authors declare that the research was conducted in the absence of any commercial or financial relationships that could be construed as a potential conflict of interest.

## Publisher’s Note

All claims expressed in this article are solely those of the authors and do not necessarily represent those of their affiliated organizations, or those of the publisher, the editors and the reviewers. Any product that may be evaluated in this article, or claim that may be made by its manufacturer, is not guaranteed or endorsed by the publisher.

## Footnotes

1
https://www.alzforum.org/alzbiomarker/ad-vs-ctrl

## Abbreviations

Aβ: amyloid beta
AD: Alzheimer’s disease
APP: amyloid beta precursor protein
AT(N): amyloid, tau, (neurodegeneration)
CNS: central nervous system
CSF: cerebrospinal fluid
DLB: dementia with Lewy bodies
DS: down syndrome
ELISA: enzyme-linked immunosorbent assay
FAD: familial Alzheimer’s disease
FTLD: frontotemporal lobe dementia
GFAP: glial fibrillary acidic protein
MCI: mild cognitive impairment
MS: mass spectrometry
NfL: neurofilament light chain
NFT: neurofibrillary tangles
PET: positron emission tomography
p-tau: phosphorylated tau
Simoa: Single molecule array
sTREM2: soluble triggering receptor expressed on myeloid cells 2
TREM2: triggering receptor expressed on myeloid cells 2.
